# MDM2 antagonists synergize broadly and robustly with compounds targeting fundamental oncogenic signaling pathways

**DOI:** 10.18632/oncotarget.1918

**Published:** 2014-04-23

**Authors:** Anne Y. Saiki, Sean Caenepeel, Dongyin Yu, Julie A. Lofgren, Tao Osgood, Rebecca Robertson, Jude Canon, Cheng Su, Adrie Jones, Xiaoning Zhao, Chetan Deshpande, Marc Payton, Jebediah Ledell, Paul E. Hughes, Jonathan D. Oliner

**Affiliations:** ^1^ Department of Oncology Research, Amgen, Thousand Oaks, CA; ^2^ Department of Biostatistics, Amgen, Seattle, WA; ^3^ Department of Molecular Structure and Characterization, Amgen, South San Francisco, CA; ^4^ Department of Molecular Sciences and Computational Biology, Amgen, Thousand Oaks, CA; ^5^ Department of Biology, Zalicus, Cambridge, MA

**Keywords:** MDM2, synergy, MEK, MAPK, PI3K, FOXM1

## Abstract

While MDM2 inhibitors hold great promise as cancer therapeutics, drug resistance will likely limit their efficacy as single agents. To identify drug combinations that might circumvent resistance, we screened for agents that could synergize with MDM2 inhibition in the suppression of cell viability. We observed broad and robust synergy when combining MDM2 antagonists with either MEK or PI3K inhibitors. Synergy was not limited to cell lines harboring MAPK or PI3K pathway mutations, nor did it depend on which node of the PI3K axis was targeted. MDM2 inhibitors also synergized strongly with BH3 mimetics, BCR-ABL antagonists, and HDAC inhibitors. MDM2 inhibitor-mediated synergy with agents targeting these mechanisms was much more prevalent than previously appreciated, implying that clinical translation of these combinations could have far-reaching implications for public health. These findings highlight the importance of combinatorial drug targeting and provide a framework for the rational design of MDM2 inhibitor clinical trials.

## INTRODUCTION

Cancer therapies targeting tumor-specific genetic alterations have shown clinical promise, in some cases eliciting remarkable tumor regressions and accompanying improvements in survival [1]. However, these therapeutic benefits are rarely durable. Following initial pharmacologic tumor debulking, rare, pre-existing subclones of drug-resistant tumor cells typically expand to repopulate the tumor, often within months of the initiation of targeted therapy. Still more challenging, many tumors fail to exhibit even an initial response to such therapies, despite harboring clonal oncogenic alterations in the genes or signaling pathways against which these agents are targeted. This form of resistance may reflect a dependency on more than one signaling axis, or in some cases overlapping signaling networks, that drive growth and survival in such tumors.

Each of the above scenarios involves a type of intrinsic resistance, irrespective of whether the pre-existing drug insensitivity involves all or just a few cells within the tumor. Combating such resistance will undoubtedly require treating patients with drug combinations [1-3]. The goals of such combinations will be 1) to eliminate rare cells that are resistant to targeted single agents before they can expand and 2) to simultaneously suppress cooperatively acting oncogenic driver signals.

Loss of p53 activity constitutes one of the most important drivers of oncogenesis. p53 is a tumor suppressor and transcription factor that responds to cellular stress by activating the transcription of numerous genes involved in cell cycle arrest, apoptosis, senescence, and DNA repair [4]. Unlike normal cells, which have infrequent cause for p53 activation, tumor cells are under constant cellular stress from various insults including hypoxia and pro-apoptotic oncogene activation. Thus, there is a strong selective advantage for inactivation of the p53 pathway in tumors, and it has been proposed that eliminating p53 function may be a prerequisite for tumor survival [5]. In support of this hypothesis, three groups of investigators have used mouse models to demonstrate that absence of p53 function is a continuous requirement for the maintenance of established tumors [6-8]. When the investigators restored p53 function to tumors with inactivated p53, the tumors regressed. More recent preclinical work by two of these groups indicated that this regression might be specific to higher-grade tumors [9, 10].

p53 is inactivated by mutation and/or loss in approximately 50% of tumors [11]. Other key members of the p53 pathway are also genetically or epigenetically altered in cancer. MDM2, an oncoprotein, inhibits p53 function, and it is activated by gene amplification at high frequency in sarcomas and at low frequency in cancers of the brain, bladder, stomach, lung, skin, and breast [11]. MDM2, in turn, is inhibited by another tumor suppressor, ARF. It has been suggested that alterations downstream of p53 may be responsible for at least partially inactivating the p53 pathway in p53^WT^ tumors. In support of this concept, some p53^WT^ tumors appear to exhibit reduced apoptotic potential, although their ability to undergo cell cycle arrest remains intact [12].

The ability to activate p53 in human tumors has been a long-standing and elusive therapeutic goal, but significant advances towards this objective have been made in the last several years [13, 14]. The most promising treatment strategy identified to date involves use of small molecules that bind MDM2 and neutralize its interaction with p53. MDM2 inhibits p53 activity by three mechanisms: 1) acting as an E3 ubiquitin ligase to promote p53 degradation, 2) binding to and blocking the p53 transcriptional activation domain, and 3) exporting p53 from the nucleus to the cytoplasm [4]. All three of these mechanisms can be blocked by neutralizing the MDM2-p53 interaction. This therapeutic strategy could potentially be applied to the roughly 50% of tumors that are p53^WT^, and preclinical studies with small molecule MDM2 inhibitors have yielded promising reductions in tumor growth both *in vitro* and *in vivo* [13, 14].

Nonetheless, even in p53^WT^ tumors, single-agent MDM2 inhibition is unlikely to confer dramatic and durable inhibition of tumor growth in the majority of cancer patients. It is clear that MDM2 inhibition can drive the selective expansion of rare p53-inactivated tumor cells [8, 15], and additional agents will have to be co-administered to eliminate such cells. Furthermore, both cultured tumor cells and human tumors show variable initial responses to MDM2 inhibitors [12, 16-18], and it will likely be necessary to inhibit other survival signals to unmask the full apoptotic potential of p53 activation.

Towards the goal of preempting resistance to MDM2 inhibition and eliciting long term disease control, a cell-based screen was conducted to identify compounds that might synergize with MDM2 inhibitors in the inhibition of tumor cell viability. Among the top screening hits were compounds targeting fundamental oncogenic pathways, including the PI3K and MAPK pathways, thus providing possible combinations to evaluate in clinical trials.

## RESULTS

### Combination Screening Revealed Compounds that Synergize with MDM2 Inhibitors

**Figure 1:**
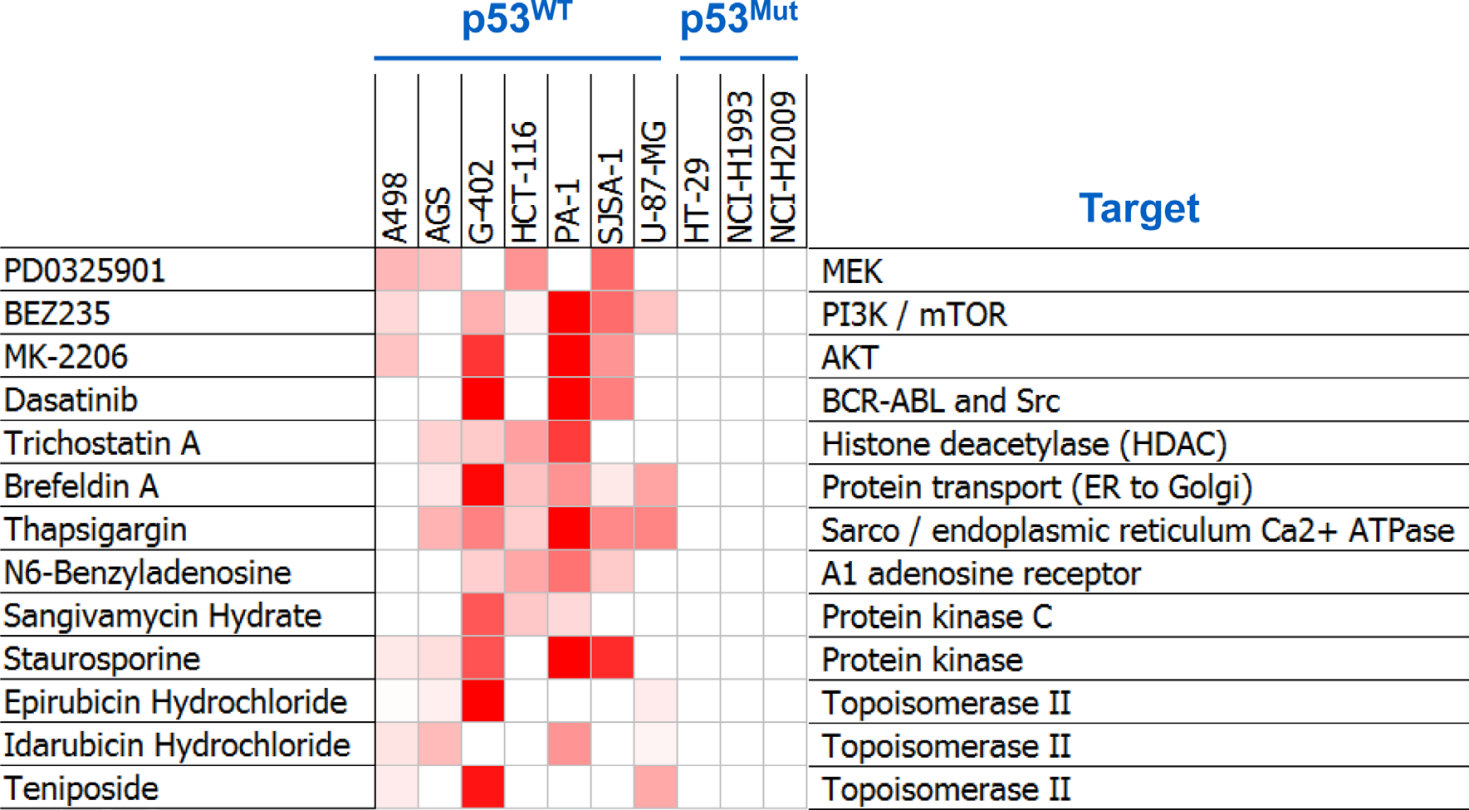
Combination Screening Yielded Hits Exhibiting p53-Dependent Synergy with MDM2 Inhibition Heat-map representation of synergy scores from the thirteen compounds shown to synergize with MDM2 inhibition. Cell viability was assessed by ATP quantification following 72 hours of inhibitor treatment. Synergy scores were calculated using the Loewe additivity model. Darker red indicates greater synergy. See also Tables S1-S3.

To identify agents that might synergize with MDM2 inhibition in the suppression of cell viability, 1169 compounds targeting a diverse array of mechanisms (Table S1) were screened in pair-wise combinations with an MDM2 inhibitor called C-25 [19] (Table S2) across ten cell lines (seven p53^WT^ and three p53^Mutant^). The p53^Mutant^ cell lines served as negative controls, as no synergy would be expected in cell lines that lack the capacity to respond to single-agent MDM2 inhibition. A combination was called as a hit in this screen when ≥ 3 of the seven p53^WT^ cell lines (but none of the three p53^Mutant^ cell lines) displayed synergy, as determined using the Loewe additivity model [20]. In total, thirteen of the 1169 library compounds (1.1%) exhibited synergy with the MDM2 inhibitors (Figure 1). Remarkably, three of the 13 screen hits were compounds targeting the MAPK and PI3K pathways (PD0325901, a MEK kinase inhibitor; BEZ235, a dual PI3K/mTOR kinase inhibitor; and MK-2206, an AKT kinase inhibitor).

**Figure 2:**
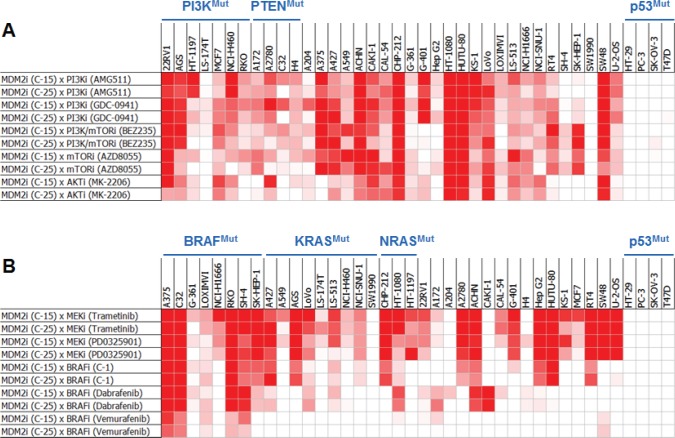
Pair-wise Combinations of MDM2 Inhibitors with PI3K or MAPK Pathway Inhibitors Exhibit Broad and Robust Synergy Across Cell Lines Heat-map representation of synergy scores from MDM2 inhibitor combinations with (A) PI3K or (B) MAPK pathway inhibitors across a panel of 40 cell lines. Cell viability was assessed by ATP quantification following 72 hours of inhibitor treatment. Synergy scores were calculated using the Loewe additivity model. Darker red indicates greater synergy. See also Tables S2-S4.

To confirm these 3 hits and determine how broadly these synergies might extend across tumor cell types, an independent set of 40 cell lines (thirty-six p53^WT^ and four p53^Mutant^) was screened with these compounds (Table S3). Additional compounds targeting the PI3K and MAPK pathways were also profiled in this screen 1) to determine whether intervention at other nodes in the PI3K and MAPK pathways might also synergize with MDM2 inhibition, 2) to dissect the individual roles of PI3K and mTOR inhibition in the BEZ235-mediated synergy, and 3) to ensure that the synergy conferred by the primary screening hits targeting the PI3K and MAPK biochemical axes was pathway-specific, rather than compound-specific (Table S4). The additional compounds included in this follow-up screen included a MEK inhibitor (trametinib), three BRAF inhibitors (dabrafenib, vemurafenib, and a preclinical-stage compound called C-1 [21]), two PI3K inhibitors (AMG 511 and GDC-0941), and an mTOR kinase inhibitor (AZD8055). Several striking findings were identified in this screen (Figure 2). First, combinations of MDM2 antagonists and PI3K pathway inhibitors exhibited broad and robust synergy, irrespective of which node in the PI3K pathway was targeted; furthermore, the synergy was not limited to cell lines harboring PI3K pathway mutations (Figure 2A). Similarly, combinations of MDM2 and MEK inhibitors displayed strong and widespread synergy, independent of MAPK pathway mutational status (Figure 2B). BRAF inhibitors also synergized with MDM2 inhibitors, and the greatest cooperativity was seen in the BRAF^Mutant^ cell lines (Figure 2B).

A third screen, performed with the clinical-stage MDM2 inhibitor, AMG 232, confirmed the breadth of synergy with PI3K and MAPK pathway antagonists and extended this finding to hematologic tumor cell lines (Figure S1). This screen was designed and powered to enable a statistical assessment of synergy for each compound combination across all cell lines in which it was tested. All combinations were performed using four biological replicates, each of which was run in technical duplicate. A heterologous compound combination (AxB) was considered synergistic only when its synergy score was statistically greater than those of both its cognate self-crosses (AxA and BxB), as determined using a Loewe additivity model. In the twenty p53^W^T cell lines tested, 49% of the AMG 232 x PI3K pathway inhibitor combinations and 76% of the AMG 232 x MEK inhibitor combinations exhibited synergy (Figure S1). In the five p53^WT^ cell lines harboring BRAF mutations, 75% of the AMG 232 x BRAF inhibitor combinations displayed synergy.

### MDM2 Inhibitor Combinations with PI3K or MEK Inhibitors Yielded Dramatic Increases in Apoptosis but Small Incremental Reductions in Proliferation

**Figure 3:**
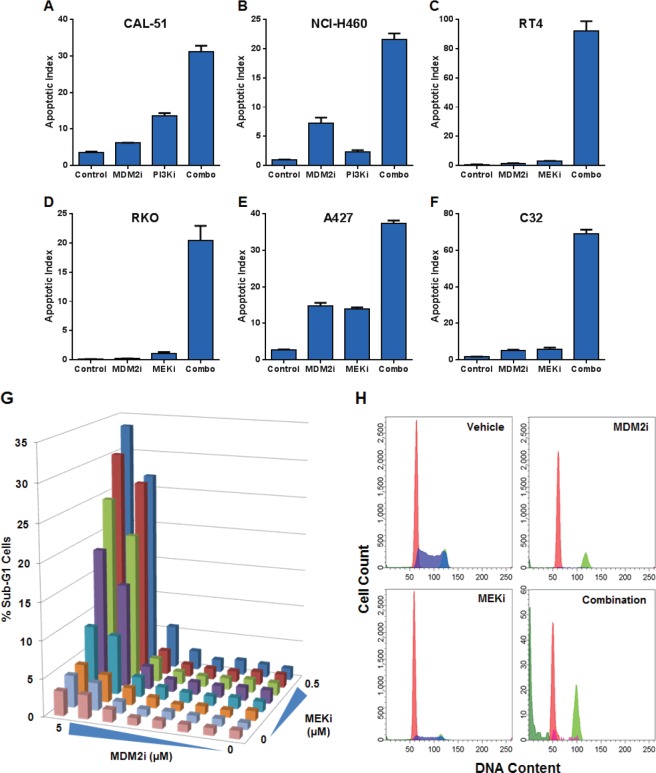
MDM2 Inhibitor Combinations with PI3K and MEK Inhibitors Yield Dramatic Increases in Apoptosis but Small Incremental Reductions in Proliferation (A) CAL-51 or (B) NCI-H460 cells were treated with DMSO (control), 3 μM C-15 (MDM2 inhibitor), (A) 0.3 μM or (B) 3 μM AMG 511 (PI3K inhibitor), or a combination of C-15 plus AMG 511 in the presence of caspase 3/7 substrate for (A) 8 hours or (B) 48 hours. (C) RT4, (D) RKO, (E) A427 or (F) C32 cells were treated with DMSO (control), 3 μM C-15 or (C) 0.3 μM or (D-F) 3 μM trametinib (MEK inhibitor) for (D) 30 hours or (C, E, F) 48 hours as described above. Apoptotic indices were calculated as the percentage of caspase-positive objects relative to the total number of DNA-containing objects; mean and SEM (n=3) are shown. RKO cells were treated with a dose titration matrix (3-fold dilution series) of C-15 (MDM2i) and trametinib (MEKi) for 48 hours and pulsed with bromodeoxyuridine prior to the end of treatment. Cells were trypsinized, fixed, permeabilized, acid-treated, and stained with propidium iodide, anti-BrdU and anti-caspase-3 antibodies. (G) The percentage of sub-G1 cells was measured by flow cytometry for each condition in the dose titration matrix. (H) Representative DNA ploidy plots for cells treated with vehicle, 5 μM C-15, 0.5 μM trametinib, or a combination of C-15 plus trametinib with populations of G1 (red), S (blue), G2/M (light green), sub-G1 (dark green), and caspase-3 positive (magenta) cells shown.

**Figure 4:**
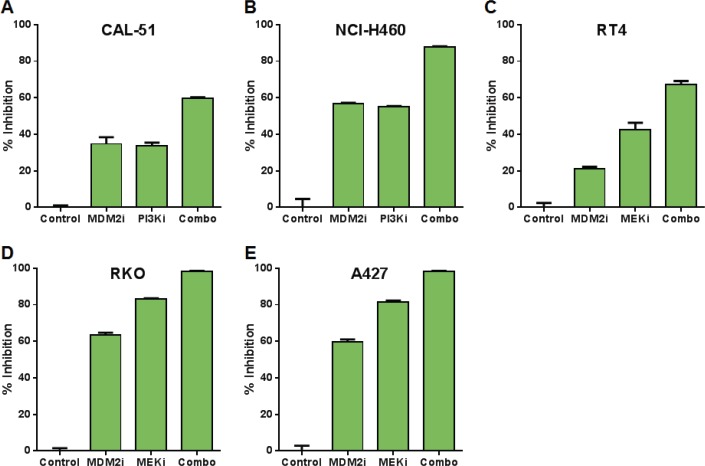
Combinations of MDM2 and PI3K or MEK Inhibition Produce Modest Changes in Cell Proliferation Relative to Single Agents (A) CAL-51 or (B) NCI-H460 cells were treated with DMSO (control), 0.3 μM C-15 (MDM2 inhibitor), (A) 0.3 μM or (B) 3 μM AMG 511 (PI3K inhibitor), or a combination of C-15 plus AMG 511 for 24 hours and pulsed with bromodeoxyuridine prior to the end of treatment. (C) RT4, (D) RKO or (E) A427 cells were treated with DMSO (control), (C) 0.3 μM or (D, E) 1 μM C-15, or (C) 0.01 μM or (D, E) 3 μM trametinib (MEK inhibitor) as described above. Cells were stained with anti-BrdU antibody, and the percentage of BrdU-positive cells was measured by flow cytometry. Percent inhibition was calculated relative to DMSO control; mean and SEM (n=3) are shown.

Compound-mediated reductions in cell numbers could be caused by increased cell death and/or decreased cell proliferation. We assessed the possible role of each of these processes in the synergy between MDM2 inhibitors and PI3K or MAPK pathway antagonists. These studies measured caspase 3/7 activity (apoptosis), sub-G1 DNA content (death), and BrdU incorporation (proliferation). Combining MDM2 and PI3K inhibitors in the CAL-51 (breast adenocarcinoma) and NCI-H460 (non-small cell lung cancer) cell lines yielded profound inductions of apoptosis (Figure 3A-B). The same conclusions were drawn from combinations of MDM2 and MEK inhibitors in the RT4 (bladder cancer), RKO (colorectal cancer), A427 (lung cancer), and C32 (melanoma) cell lines (Figure 3C-F). Flow cytometry-based sub-G1 cell cycle analysis of the RKO cell line revealed strong synergistic cell killing in the MDM2 plus MEK inhibitor combination (Figure 3G-H). In contrast to the results of the cell death assays, BrdU incorporation studies showed substantial single-agent activity with the MDM2 and MEK inhibitors, but modest cooperativity when combined, suggesting that apoptosis induction was the dominant factor driving synergistic reductions in cell numbers (Figure 4).

### Combined Inhibition of MDM2 and MEK Elicited Synergy-Associated Changes in the Expression of Genes Modulated by FOXM1

**Figure 5:**
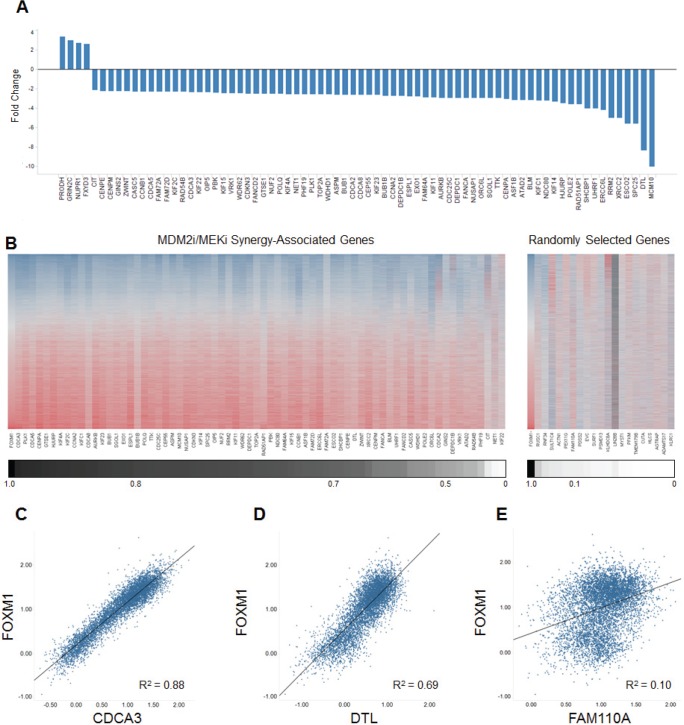
Combining MDM2 and MEK Inhibition Yields a Profile of Expression Changes that Overlaps the FOXM1 Transcriptional Target Signature (A) Fold changes in expression of the 74 MDM2i/MEKi synergy-associated genes common to both the RKO and A427 cell lines. Synergy-associated genes were defined as those whose expression increased or decreased by 1) 3.75-fold when comparing the combination group to the DMSO control and 2) 2.2-fold when comparing the combination group to both single-agent groups (synergy-associated genes had to meet both criteria). (B) Heat-map representation of the expression correlation between FOXM1 and the 70 downregulated MDM2i/MEKi synergy-associated genes or 20 unrelated randomly selected genes. Heat map reflects RNAseq RPKM expression values obtained from 6466 TCGA primary tumor samples of diverse tumor origin (20 indications). Lower scale reports coefficient of determination (R^2^) values for pair-wise linear regression analysis comparing FOXM1 expression to each of the 70 MDM2i/MEKi synergy-associated genes or 20 unrelated randomly selected genes. (C-E) Scatterplots for FOXM1 and synergy-associated genes (C) CDCA3 and (D) DTL or randomly selected gene (E) FAM110A. Scatterplots report RNAseq log_10_ RPKM expression values for 6466 TCGA primary tumor samples of diverse tumor origin (20 indications)

To investigate the molecular mechanisms underlying the observed synergy between MDM2 and MEK inhibition, we performed a global expression screen to identify genes whose mRNA levels were altered when co-targeting MDM2 and MEK. Two cell lines of different origins, RKO and A427, were selected to enable tissue-specific expression changes to be filtered out of the analysis. Each cell line was treated with DMSO (negative control), a MEK inhibitor, an MDM2 inhibitor, or both. Synergy-associated genes were defined as those whose expression increased or decreased by 1) 3.75-fold when comparing the combination group to the DMSO control and 2) 2.2-fold when comparing the combination group to both single-agent groups. Seventy-four genes (4 upregulated and 70 downregulated) met both of these criteria in both cell lines (Figure 5A). The 70 downregulated transcripts largely represented genes associated with cell division, DNA damage and repair, and apoptosis. Strikingly, 55 of these 70 genes (79%) had previously been identified as transcriptional targets of the FOXM1 transcription factor [22, 23], suggesting the possibility that suppression of FOXM1 activity might play an important role in the synergy mediated by MDM2 and MEK inhibition.

To further explore this association, we performed co-expression analysis comparing mRNA levels of FOXM1 to those of the 70 synergy-associated downregulated transcripts in 6466 primary tumor samples of diverse tissue origins [11]. This analysis revealed strong co-expression of FOXM1 with 96% of these 70 genes (Figure 5B). As a control, 20 random genes were selected for FOXM1 co-expression analysis, and none of these transcripts showed a correlation with FOXM1 expression (Figure 5B). The data in Figure 5B have also been graphed as scatterplots for two synergy-associated genes (Figure 5C-D), pointedly illustrating the very tight correlation between their expression and that of FOXM1. In contrast, no scatterplot correlation was apparent between the expression of FOXM1 and a representative randomly selected gene (Figure 5E).

It has been reported that p53 activation downregulates FOXM1 expression and that MAPK pathway inhibition blocks FOXM1 nuclear translocation [24, 25]. Conversely, p53 gene silencing has been shown to increase FOXM1 expression [24, 26]. FOXM1 has also been shown to increase the resistance of cancer cells to apoptosis, and FOXM1 knockdown re-sensitizes tumor cells to programmed cell death [27]. Together, our findings and the published data are consistent with the hypothesis that MDM2 and MEK inhibition play complementary roles in the suppression of FOXM1, resulting in synergistic induction of apoptosis.

### *In vivo* Efficacy Studies Recapitulated the *in vitro* Cooperativity Observed with Pair-wise Combinations of MDM2 Inhibitors and MAPK Pathway Inhibitors

**Figure 6:**
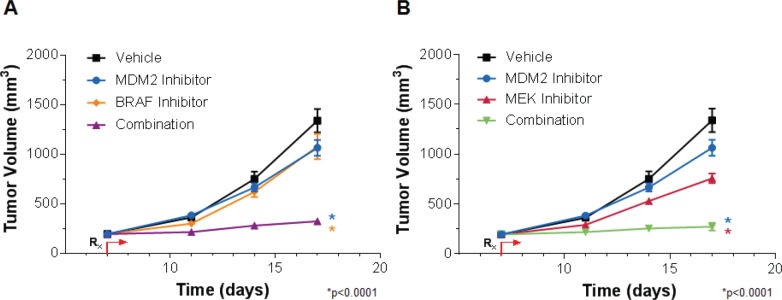
MDM2 Inhibition and MAPK Pathway Inhibition Cooperatively Suppress RKO Tumor Xenograft Growth (A) RKO tumor-bearing mice were treated with vehicle, 100 mg/kg AMG 232 (MDM2 inhibitor) or 10 mg/kg C-1 (BRAF inhibitor) alone or in combination, or with (B) vehicle, 100 mg/kg AMG 232 or 10 mg/kg PD0325901 (MEK inhibitor) alone or in combination. Data are represented as mean tumor volumes, and error bars represent SEM (n=10 mice/group). *p < 0.0001 combination groups vs. single agents by factorial RMANOVA followed by Dunnett's post hoc analysis for repeated measurements.

To assess whether the cooperative actions of MDM2 inhibition and MEK or BRAF inhibition seen *in vitro* could be translated to the *in vivo* setting, these agents were orally administered in a pair-wise fashion to mice harboring human RKO colon tumor xenografts (Figure 6). The RKO model was chosen for this purpose because it is particularly resistant to single agents targeting these 3 proteins. MDM2, MEK and BRAF inhibitor monotherapy conferred tumor volume reductions of 24, 51 and 23%, respectively. But in combination, MDM2 plus BRAF inhibition (Figure 6A) and MDM2 plus MEK inhibition (Figure 6B) elicited 89 and 93% decreases in tumor volume, respectively (p < 0.0001 for each combination vs. its respective single-agent comparators). There were no adverse effects on body weight in any of the treatment groups. However, it was not possible to draw conclusions about MDM2 inhibitor safety from these studies, as this series of MDM2 antagonists displays very weak activity against murine MDM2 (data not shown). Definitive safety assessment awaits evaluation of these combinations in humans. A second point not addressed by this study is the durability of response upon withdrawal of therapy. Future *in vivo* studies will evaluate this question over extended observation periods and will involve pair-wise MDM2 inhibitor combinations with MAPK and PI3K antagonists.

### Triple Combinations of MDM2, PI3K, and MEK Inhibitors Produced Greater Reductions in Cell Numbers than Two-Way Combinations of these Agents

We next evaluated whether PI3K inhibition and MEK inhibition might also synergize when combined with one another. Combining PI3K and MAPK pathway antagonists yielded synergy in a majority of the 39 cell lines tested (Figure S2). Synergy was also observed between PI3K and BRAF antagonists, and this cooperativity was enriched in BRAF-mutant cell lines (Figure S2). Together, the above results demonstrated that all three pair-wise combinations of MDM2, MAPK, and PI3K pathway inhibitors could elicit broad and robust synergy across a wide array of tumor cell lines, and they raised the possibility that triple combinations of agents targeting these key oncogenic pathways might enhance therapeutic index (the ratio between the therapeutic dose and the toxic dose) and evoke durable clinical responses.

**Figure 7:**
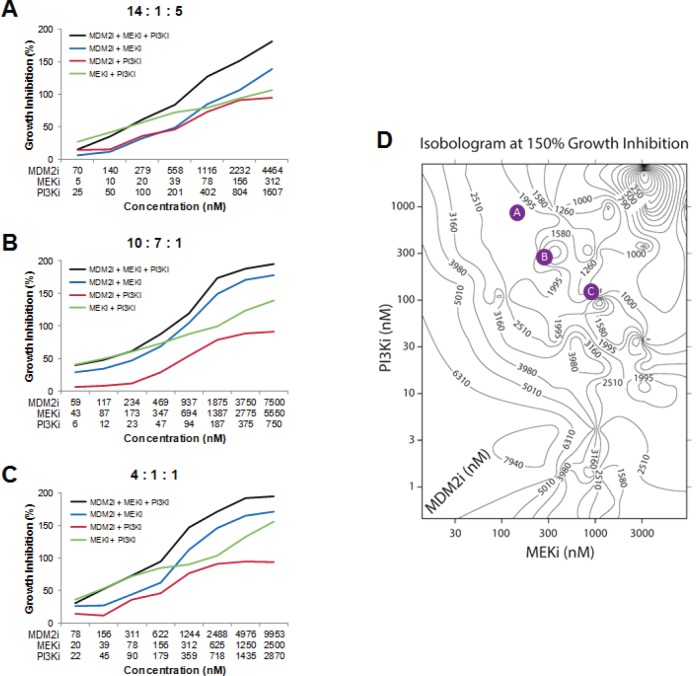
Triple Combinations of MDM2, PI3K, and MEK Inhibitors Produces Greater Reductions in Cell Viability than Two-way Combinations of these Agents (A-C) Growth inhibition curves in the RKO cell line for triple combinations of MDM2 inhibitor (AMG 232), MEK inhibitor (PD0325901), and PI3K inhibitor (AMG 511), as well as all 3 cognate double combinations. (D) Representative isobologram showing the concentrations of MDM2 inhibitor (AMG 232), MEK inhibitor (PD0325901), and PI3K inhibitor (AMG 511) that together confer 150% growth inhibition in the RKO cell line. Values on each contour indicate the concentration of MDM2 inhibitor. The molar ratios shown in A-C have been mapped onto this isobologram as colored circles. See also Table S5.

As a first step towards this goal, triple combinations of MDM2, PI3K, and MEK inhibitors were evaluated in 6 cell lines. Each agent was evaluated over a 10-point exposure-response curve in combination with the other two agents to create 10 x 10 x 10 cuboidal matrices. Growth inhibition at each 3-way combination data point in the cube was then compared to the growth inhibition conferred by the three 2-way combinations that used the same compound concentrations as in the triple combination. In so doing, it was possible to identify regions within each cube in which the triple combination was statistically superior to all of its cognate double combinations. To determine whether these regions might harbor features common to all 6 cell lines, we developed algorithms to investigate whether specific ratios of the three agents could provide universally superior anti-proliferative activity. Two such MDM2:MEK:PI3K inhibitor molar ratios were identified (Table S5). These two triple combination ratios conferred enhanced growth suppression relative to their three constituent double combination ratios over broad concentration ranges (Figure 7A-B). While these represent privileged ratios, there were several additional ratios in which the 3 compounds mediated statistically greater growth inhibition than their cognate double combinations across four or five of the 6 cell lines (7 and 2 instances, respectively; Table S5). We developed another algorithm to identify ratios of the 3 agents that maximized growth suppression irrespective of how the constituent double combinations performed. Two such combinations were identified (Table S5), one of which is exemplified in Figure 7C.

We observed that identical levels of growth inhibition could be achieved using vastly different concentration combinations for each of the 3 agents in a cuboidal matrix. To visualize this, we generated three-dimensional isobolograms identifying contours of equal effect level. Growth inhibition was represented on a 200-percentage-point scale, where 0%, 100%, and 200% indicate no effect, cell stasis, or complete cell regression, respectively. Figure 7D shows a sample isobologram at 150% growth inhibition; the specific ratios shown in Figures 7A-C have been mapped onto this surface (Figure 7D).

The agents used in these experiments target fundamental signaling pathways that are also important for the survival and proliferation of non-transformed cells. It is therefore possible that only a subset of the combinations in high-effect-level isobolograms would be clinically tolerable. In the clinic, the flexibility to titrate each agent in a combination individually would, in principal, afford the ability to optimize safety for any given efficacy level. *In vitro* studies such as these, along with human safety and efficacy data from single-agent and two-way clinical combinations, may inform the judicious selection of doses or dose ratios to evaluate in higher-order combination clinical trials.

### MDM2 Inhibitors Synergized with Agents Targeting Other Key Neoplastic Pathways and Processes

Broad and striking synergy was also observed with other MDM2 inhibitor combinations in our screens. MDM2 antagonists synergized with the dual Bcl-2/Bcl-xL inhibitors ABT-737 and ABT-263 (navitoclax) across an array of solid tumor cell lines (Figure S3A-B) and hematologic cell lines (Figure S3B). In contrast, MDM2 inhibitor synergy with the Bcl-2-selective inhibitor, ABT-199, was largely confined to hematologic cell lines, implying that Bcl-xL inhibition is required for synergy with MDM2 antagonism in most solid tumor cell lines (Figure S3B).

Imatinib, the first BCR-ABL kinase inhibitor approved for the treatment of chronic myelogenous leukemia (CML), was observed to synergize with MDM2 inhibition in two BCR-ABL-positive CML cell lines, CML-T1 and BV173 (data not shown). Four second-generation BCR-ABL inhibitors (dasatinib, nilotinib, bosutinib and ponatinib) were also tested alongside imatinib in the CML-T1 cell line and shown to synergize with MDM2 antagonism (Figure S4). Remarkably, dasatinib, which is a broad-spectrum kinase inhibitor, synergized with MDM2 inhibition in numerous solid tumor cell lines of a variety of tissue origins (Figures 1 and S5), suggesting that its clinical utility may extend beyond tumors harboring BCR-ABL fusions.

Three histone deacetylase (HDAC) inhibitors, trichostatin A, panobinostat, and mocetinostat, synergized with MDM2 inhibition in several cell lines (Figures 1 and S6). Panobinostat is under advanced clinical investigation (www.clinicaltrials.gov) and was chosen for mechanistic studies in 3 of the cell lines in which it showed strong synergy with MDM2 inhibition (A204, G401, and A2780). As was the case when combining MDM2 and either MAPK or PI3K pathway inhibitors (Figure 3), the combination of MDM2 inhibition and panobinostat produced a much higher apoptotic index than was conferred by either of the single agents alone (Figure S6C-E). And in two of these three cell lines (A204 and G401), the anti-proliferative effects of the combination were only marginally greater those of the single agents (Figure S6F-H), similar to the results seen in the pair-wise MDM2 inhibitor combinations with MAPK and PI3K pathway antagonists (Figure 4).

## DISCUSSION

As a first step towards optimizing the clinical utility of MDM2 inhibitors, we screened an 1169-compound library for agents that might synergize with MDM2 inhibition in the suppression of tumor cell viability. Through this *in vitro* screen, as well as follow-up studies, we identified several classes of compounds that displayed synergy with MDM2 antagonists. These included PI3K pathway inhibitors (targeting PI3K, mTOR, and/or AKT), MAPK pathway inhibitors (targeting MEK or BRAF), BH3 mimetics, BCR-ABL kinase antagonists, and HDAC inhibitors. We explored the mechanism of action underlying the synergies with PI3K, MEK, and HDAC inhibitors and discovered a dramatic enhancement in apoptosis in these MDM2 inhibitor combinations. Global expression analysis of cell lines treated with an MDM2 x MEK inhibitor combination yielded a set of 70 downregulated synergy-associated expression changes. Strikingly, the vast majority of these expression changes overlapped the FOXM1 transcriptional target signature, suggesting that FOXM1 was a critical mediator of the synergy conferred by concurrent modulation of these two fundamental pathways. Triple combination studies using MDM2, PI3K, and MEK or BRAF inhibitors demonstrated that even greater inhibitions of cell growth could be achieved than those conferred by each of the three 2-way combinations.

This is the first broad-based, systematic screen for compounds capable of synergizing with MDM2 antagonism. Some of the synergistic interactions described in our screens were previously identified through candidate-based approaches performed in restricted populations of tumor cell types. For example, MDM2 inhibition was shown to synergize with: 1) MAPK pathway inhibition in acute myeloid leukemia [AML] and melanoma cells [28-31], 2) PI3K pathway antagonism in AML and acute lymphoblastic leukemia cells [32, 33], 3) HDAC inhibition in AML, lung, and ovarian cells [34, 35], 4) dual Bcl-2/Bcl-xL inhibition in AML cells [36], 5) BCR-ABL kinase inhibition in Philadelphia chromosome-positive chronic myelogenous leukemia and acute lymphoblastic leukemia cells [37], and 6) dasatinib in primary B chronic lymphocytic leukemia cells [38]. Our screens revealed that synergy between MDM2 inhibitors and PI3K pathway inhibitors, MEK inhibitors, BH3 mimetics, HDAC inhibitors, and dasatinib was far more prevalent than previously appreciated, occurring across multiple tumor cell types and genetic backgrounds. These results greatly expand the potential utility of MDM2 inhibitors for treating cancer and imply that clinical translation of these combinations could have far-reaching implications for public health.

We paid special attention to the PI3K and MAPK pathways, given their central roles in oncogenesis and signal transduction. Pharmacologic intervention with kinase inhibitors targeting any level of the PI3K pathway, including PI3K itself, AKT, or mTOR, yielded quantitatively comparable synergy in combination with MDM2 inhibition. In addition, we demonstrated synergy between MEK inhibition and PI3K inhibition that extended across multiple tumor cell types. Together, these results establish a synergy triangle involving all three pair-wise combinations of MDM2, MAPK, and PI3K pathway inhibitors, and they inspire the hope that triple combinations of agents targeting these pathways might produce durable clinical responses across large patient populations.

It is clear, however, that each of these agents displays clinical side effects, as might be expected of inhibitors that target pathways that are fundamental to the growth and survival of organisms as a whole and not just their tumors [16-18, 39, 40]. Combining agents will be necessary to overcome resistance, but it will almost certainly not be possible to use the highest doses that are tolerable as single agents. Early clinical results from two-way combinations of PI3K and MAPK pathway inhibitors suggest that dual inhibition of these pathways may yield both greater efficacy and greater toxicity [41, 42], and it will very likely be necessary to dial back the dose of one or more of the inhibitors in a triple combination in order to achieve adequate tolerability. The cell-based isobole analysis described here, along with an understanding of each of the single-agent adverse event profiles, may provide a roadmap for the flexible selection of MDM2, PI3K, and MAPK pathway inhibitor dose combinations for clinical hypothesis testing. Two-way clinical combinations involving MDM2 inhibitors could provide additional data upon which to design triple combination trials.

Despite the central roles and high frequencies of PI3K, MAPK, and p53 pathway dysregulation in human cancer, it is remarkable how little clinical efficacy has been achieved with single agents targeting each of these axes [16-18, 39, 40]. With the exception of MAPK pathway inhibitors in melanoma, these agents have yielded few clinical responses, even in genetic contexts in which logic would dictate that they should. BRAF inhibitors have been largely ineffective in BRAF-mutant colorectal cancer, MEK inhibitors have had little impact in a variety of RAS-mutated tumors, and PI3K inhibitors have yielded disappointing results in tumors harboring alterations in PTEN and PI3Kα [39, 40]. Rapamycin analogs have shown efficacy, but only in restricted clinical settings [40]. Clinical data have been reported for only one MDM2 inhibitor thus far, and objective responses have been seen at low frequencies in AML and liposarcoma, but not in other p53^WT^ solid tumors [16-18].

Why have agents targeting these 3 pathways failed to produce more substantial clinical benefits? First, it may be necessary to more fully suppress PI3K or MAPK signaling or to more fully activate the p53-mediated transcriptional program—and to do so for extended durations—in order to achieve meaningful single-agent efficacy. However, such high doses and long durations may not be clinically tolerated, even with exquisitely selective inhibitors. Optimized ratios, levels, and scheduling of such drugs in combination may improve therapeutic index. Second, compensatory feedback may stimulate alternative signals for tumor growth and survival, resulting in primary resistance. For example, crosstalk between the PI3K and MAPK pathways enables mTOR inhibition to enhance ERK phosphorylation and MEK inhibition to activate AKT [43, 44], providing a rationale for dual pharmacologic suppression of these pathways. Preclinical studies have demonstrated the *in vivo* efficacy of such combinations [45-48]. Lastly, these agents may confer activity that never reaches the clinical threshold for objective response due to the counteracting and progressively dominating influence of mutationally resistant clones. Resistance-conferring genomic alterations have been documented for each of these 3 pathways [15, 49, 50]. Eliminating resistant clones before they have the opportunity to expand will be a critical goal of orthogonal combination therapy.

Full realization of the potential of drug combinations will require paradigmatic changes in the oncology drug development process, and this evolution has begun. While it has been conventional practice to evaluate new investigational agents as monotherapies or in combination with approved standard-of-care drugs, the FDA has begun encouraging industry to co-develop compelling combinations of two or more new experimental agents [51]. Many such trials are ongoing, including those simultaneously targeting the MAPK and PI3K pathways [41, 42]. Simultaneous targeting is critical, as sequential administration of anti-cancer therapies has been shown to foster resistance. Currently, drug development and clinical practice are centered around sequential lines of therapy, and a move towards upfront concurrent combinations would be expected to yield superior efficacy [1, 2].

Additional hurdles exist to the use of combinations for achieving high-impact therapeutic outcomes. As mentioned above, a key concern is that increased antitumor efficacy might be associated with commensurate increases in toxicity to normal cells, thus leaving the therapeutic index unchanged and providing no net benefit to cancer patients. Pathway addiction has been shown to produce greater loss of viability in tumor cells than normal cells exposed to individual therapeutic interventions targeting such pathways. In principle, synergistic drug combinations targeting more than one such signaling axis might further enhance tumor:normal sensitivity, yielding increased therapeutic indices at tolerable drug levels. This hypothesis is now being tested clinically, and successes of this approach will likely incentivize further clinical exploration of synergistic combinations. Another hurdle is that drug combinations can be expensive—particularly those involving targeted therapeutics—and substantially better clinical benefit will be required to justify these higher costs. Lastly, tumor biology will dictate which agents must be combined to achieve durable clinical responses. This understanding is increasingly driving new partnerships between pharmaceutical companies with complementary drug pipelines. While collaborative co-development of combinations can present logistical and commercial challenges, achieving greater therapeutic success will frequently mandate it.

## METHODS

### Ethics Statement

All animal experimental procedures were conducted in accordance with the guidelines of the Amgen Animal Care and Use Committee and the Association for Assessment and Accreditation of Laboratory Animal Care standards.

### Synergistic Combination Studies with MDM2 Antagonists

Two-way combinations were performed and analyzed as previously described [20]. Detailed methods for two-way and three-way combinations are provided in the Supplemental Experimental Procedures.

### Measurement of Apoptotic Index

Cultured cells were treated with pharmacologic inhibitors or DMSO negative control. Apoptotic cells were labeled with 1 μM CellPlayer Kinetic Caspase-3/7 reagent (Essen Bioscience) and visualized with IncuCyte FLR (Essen Bioscience) live-cell imaging. To obtain cell number, cells were stained with Vybrant® DyeCycle™ Green Stain (Life Technologies). Apoptotic indices were calculated as the number of caspase-positive objects normalized to the total number of DNA-containing objects and reported as a percentage.

### Flow Cytometry

For cell cycle analysis, RKO cells were treated in quadruplicate 96-well plates with a dose titration matrix of C-15 and trametinib for 48 hours and pulsed with bromodeoxyuridine (BrdU) two hours prior to the end of treatment. Cells were trypsinized, fixed, permeabilized, acid-treated, and stained with anti-BrdU Alexa Fluor®647 (Life Technologies) and anti-caspase-3-FITC (BD Pharmingen) antibodies, followed by DNA staining with propidium iodide. Flow cytometry analysis was performed on an LSR II (BD Biosciences). For BrdU proliferation assays, cells were treated with DMSO or inhibitors as single agents or in combination for 24 hours. Samples were processed for BrdU incorporation as described above.

### Gene Expression Analysis

RKO and A427 cells were treated with DMSO (control), 3 μM C-15, 3 μM trametinib, or a combination of C-15 plus trametinib for 24 hours. DNA-free total RNA was extracted from RKO (n=5) and A427 (n=3) cells using the Qiagen RNeasy Plus Mini Kit (Qiagen). RNA quality was analyzed using the Agilent RNA 6000 Nano Kit (Agilent Technologies) on an Agilent 2100 Bioanalyzer (Agilent Technologies). RNA samples with an RNA integrity number (RIN) > 8 were considered acceptable. The concentration of RNA was measured on a NanoDrop 8000 spectrophotometer (Thermo Scientific). Gene expression was measured by microarray using single-color Agilent Human Whole Genome 4x180K v2 custom arrays (Agilent). Gene expression data were normalized using the quantile normalization method within ArrayStudio v6.2.2.30 (OmicSoft).

For FOXM1 co-expression analysis, RNAseq RPKM values for FOXM1, the 70 down-regulated MDM2/MEK synergy-associated genes, and 20 randomly selected genes were obtained from the TCGA for 6466 primary tumor samples encompassing 20 tumor indications. Pair-wise linear regression analysis was performed to determine the strength of association between FOXM1 and each of these genes. Genes were classified as FOXM1-coexpressed if their FOXM1 expression correlations (R^2^ values) were at least 3 standard deviations above the mean R^2^ value for the 20 randomly selected genes.

### *In vivo* Pharmacology

*In vivo* efficacy studies were performed using standard methods. Detailed methods are provided in the Supplemental Experimental Procedures.

## SUPPLEMENTARY FIGURES AND TABLES




