# Supplementary material for: MDM2 antagonists synergize broadly and robustly with compounds targeting fundamental oncogenic signaling pathways
Source: Oncotarget. 2014 Apr 23;5(8):2030–43. doi: 10.18632/oncotarget.1918 (PMC4039142; doi:10.18632/oncotarget.1918)
Supplement: Supplementary file 1 [file oncotarget-05-2030-s001.pdf]

MDM2 antagonists synergize broadly and robustly with compounds targeting fundamental oncogenic signaling pathways

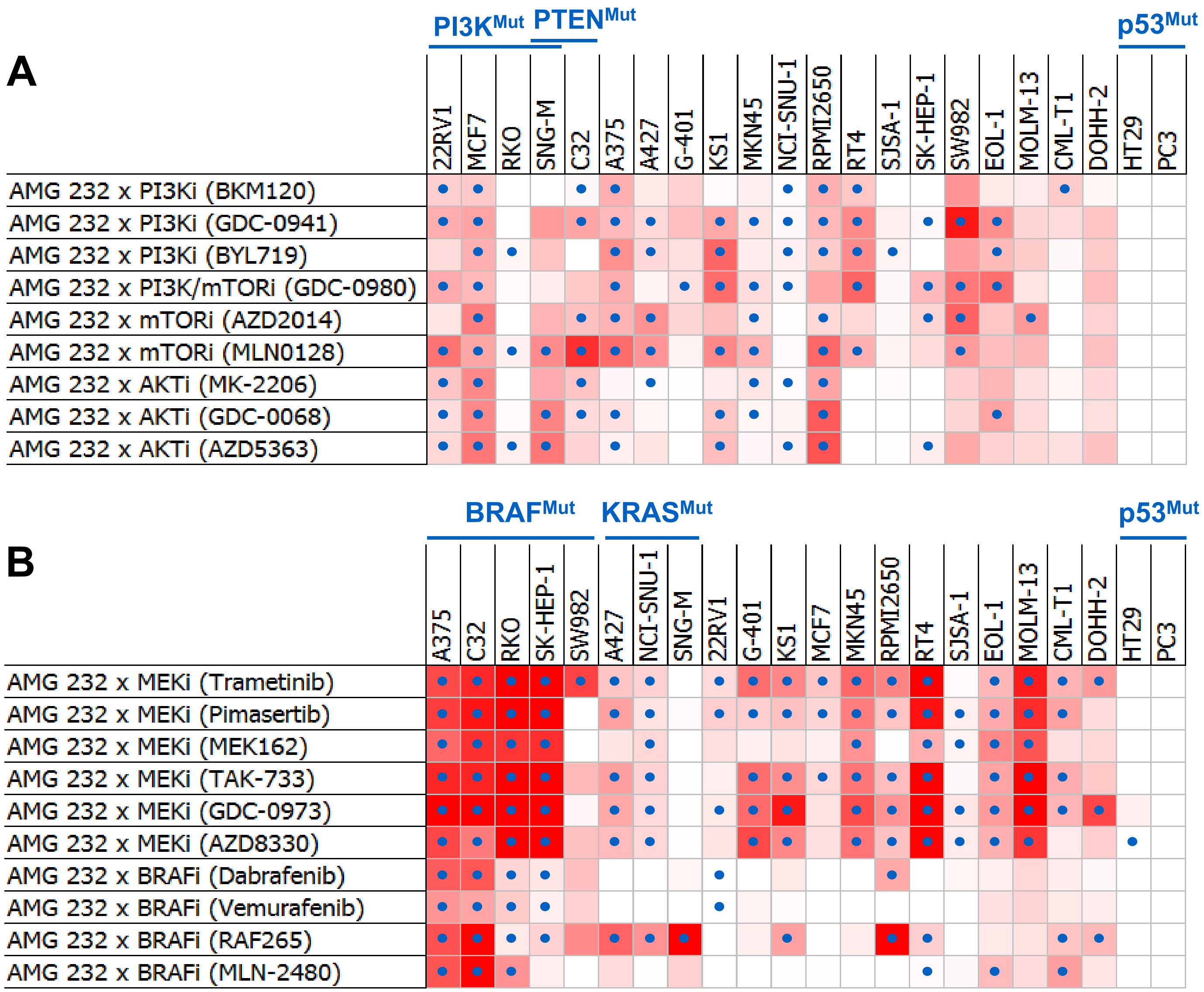

Figure S1. Heat-map representation of synergy scores from MDM2 inhibitor combinations with (A) PI3K or (B) MAPK pathway inhibitors across a panel of 22 cell lines. Cell viability was assessed by ATP quantification following 72 hours of inhibitor treatment. Synergy scores were calculated using the Loewe additivity model. Darker red indicates greater synergy. Filled blue circles denote combinations with statistically significant synergy scores.

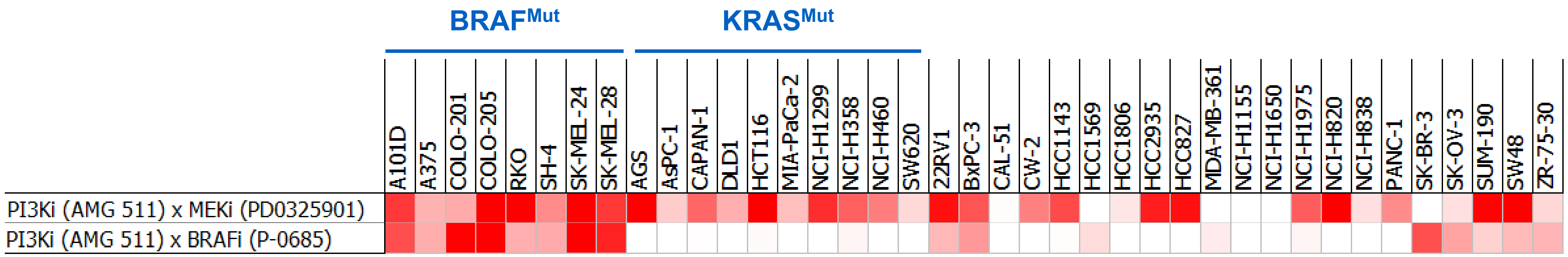

Figure S2. Heat-map representation of synergy scores from PI3K inhibitor combinations with PD0325901 (MEK inhibitor) or P-0685 (BRAF inhibitor) across a panel of 39 cell lines. Cell viability was assessed by ATP quantification following 72 hours of inhibitor treatment. Synergy scores were calculated using the Loewe additivity model. Darker red indicates greater synergy.

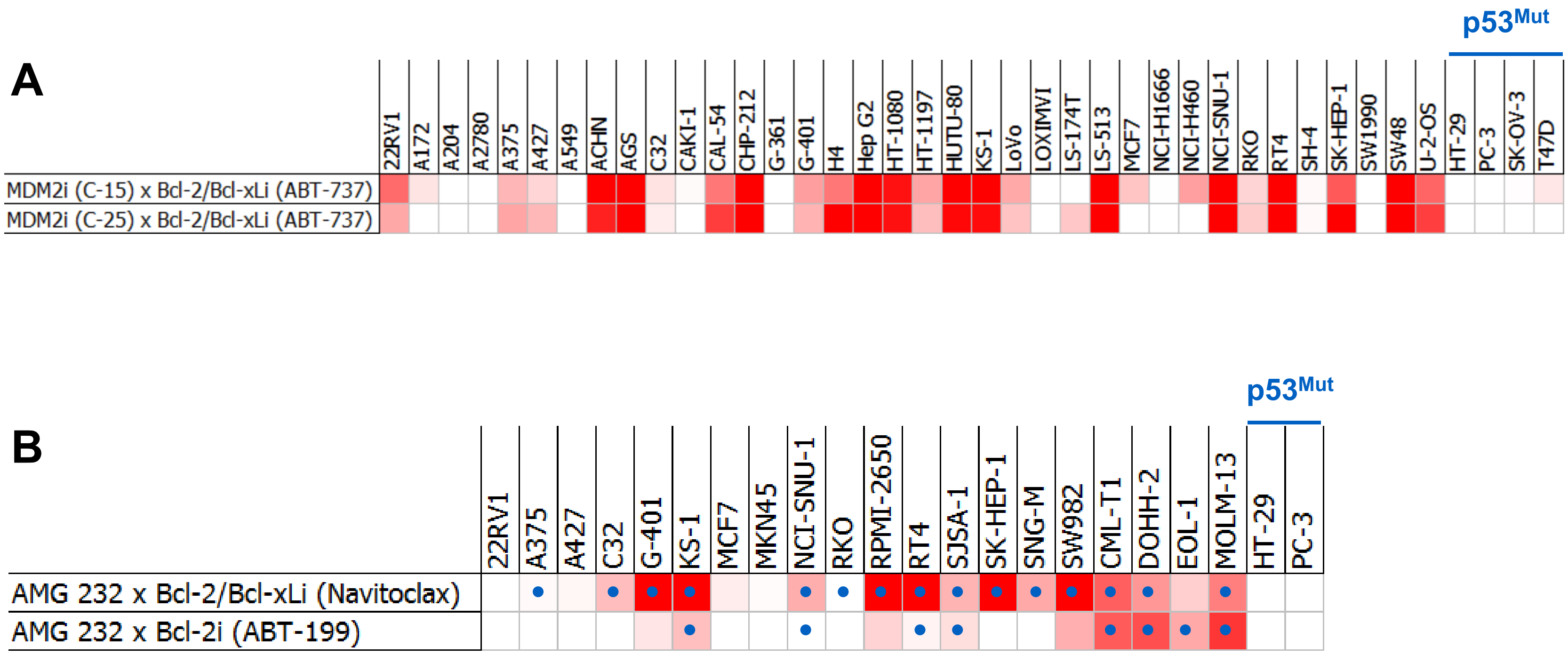

Figure S3. Heat-map representation of synergy scores from MDM2 inhibitor combinations with BH3 mimetics across panels of (A) 40 or (B) 22 cell lines. Cell viability was assessed by ATP quantification following 72 hours of inhibitor treatment. Synergy scores were calculated using the Loewe additivity model. Darker red indicates greater synergy. Filled blue circles denote combinations with statistically significant synergy scores.

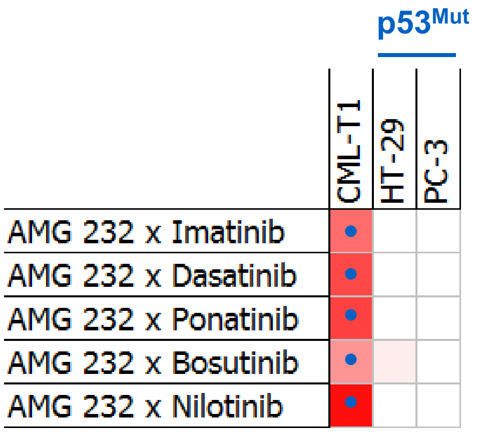

Figure S4. Heat-map representation of synergy scores from MDM2 inhibitor combinations with BCR-ABL kinase inhibitors in $p53^{WT}$ CML-T1, $p53^{Mutant}$ HT-29 or PC-3 cell lines. Cell viability was assessed by ATP quantification following 72 hours of inhibitor treatment. Synergy scores were calculated using the Loewe additivity model. Darker red indicates greater synergy. Filled blue circles denote combinations with statistically significant synergy scores.

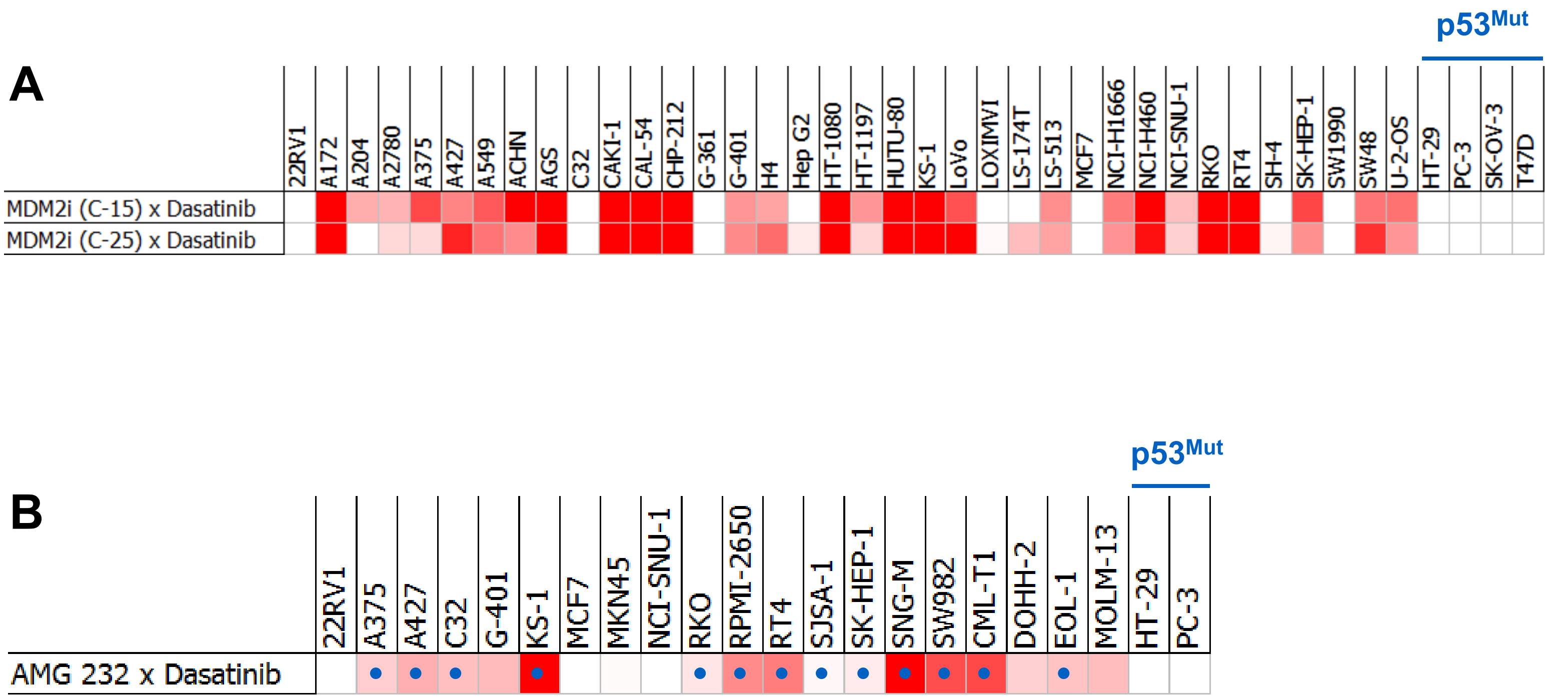

Figure S5. Heat-map representation of synergy scores from MDM2 inhibitor combinations with dasatinib across panels of (A) 40 or (B) 22 cell lines. Cell viability was assessed by ATP quantification following 72 hours of inhibitor treatment. Synergy scores were calculated using the Loewe additivity model. Darker red indicates greater synergy. Filled blue circles denote combinations with statistically significant synergy scores.

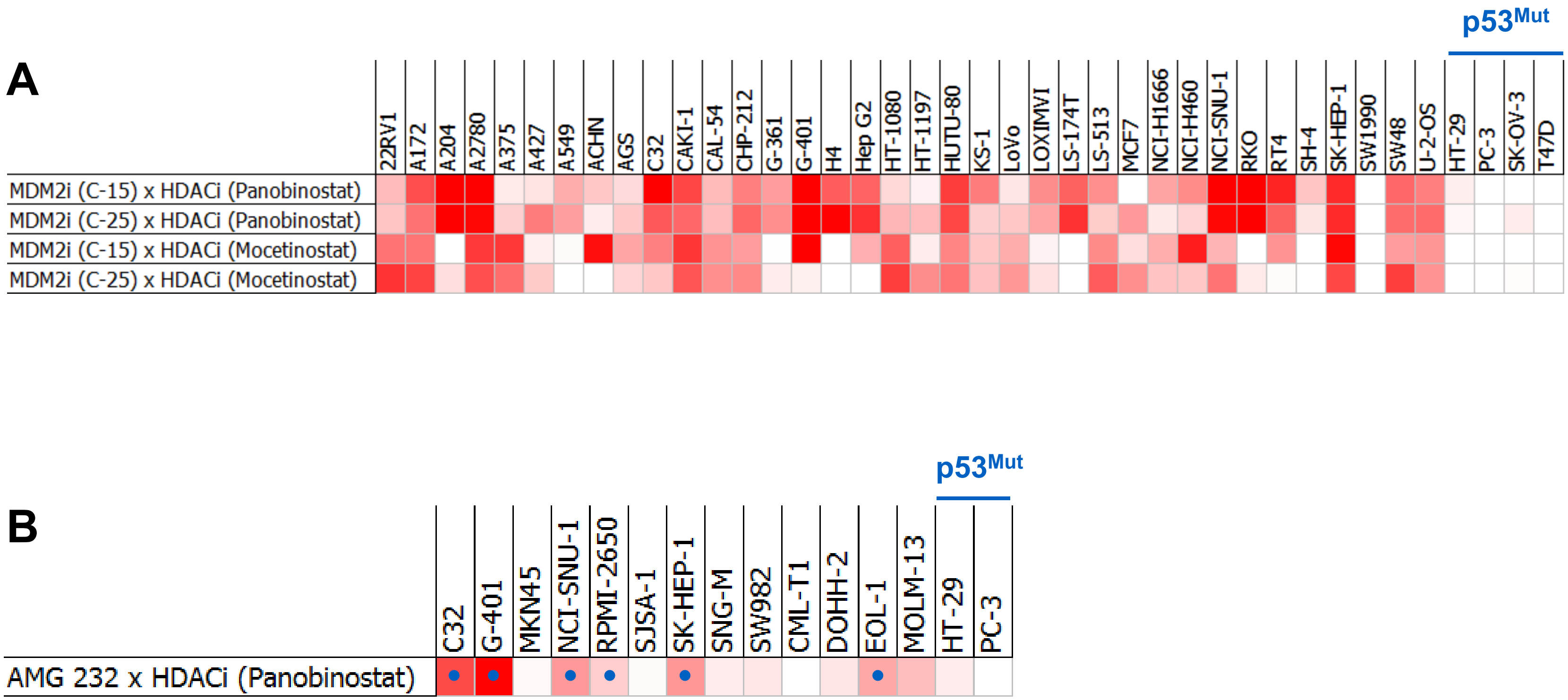

Figure S6A-B. Heat-map representation of synergy scores from MDM2 inhibitor combinations with histone deacetylase (HDAC) inhibitors across panels of (A) 40 or (B) 15 cell lines. Cell viability was assessed by ATP quantification following 72 hours of inhibitor treatment. Synergy scores were calculated using the Loewe additivity model. Darker red indicates greater synergy. Filled blue circles denote combinations with statistically significant synergy scores.

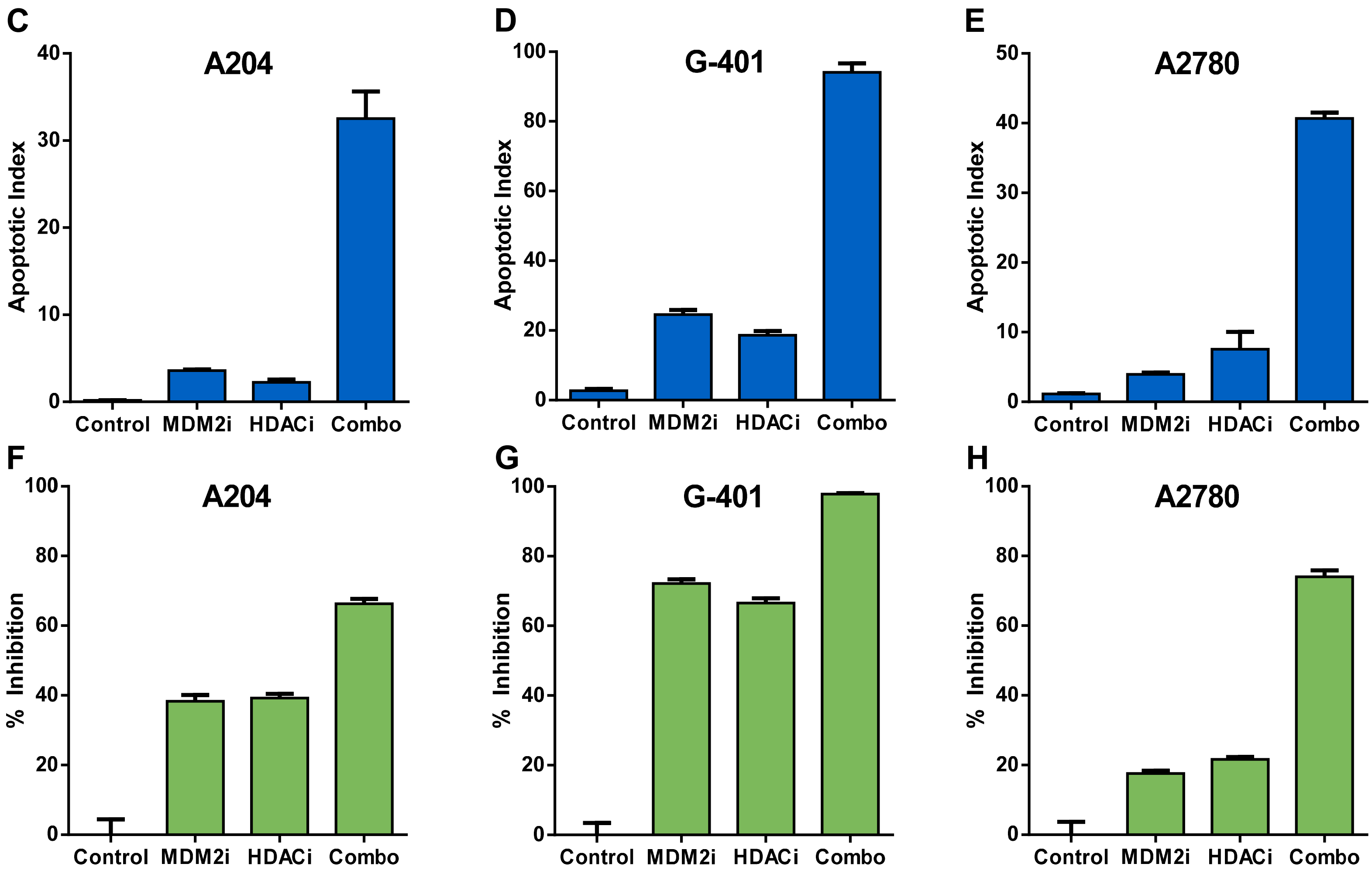

Figure S6C-H. (C) A204, (D) G-401, or (E) A2780 cells were treated with DMSO (control), (C-D) 1 μM or (E) 0.3 μM C-15 (MDM2 inhibitor), (C-D) 0.1 μM or (E) 0.03 μM panobinostat (HDAC inhibitor), or a combination of C-15 plus panobinostat in the presence of caspase 3/7 substrate for 48 hours. Apoptotic indices were calculated as the percentage of caspase-positive objects relative to the total number of DNA-containing objects; mean and SEM (n=3) are shown.

(F) A204, (G) G-401, or (H) A2780 cells were treated with DMSO (control), (F) 0.1 μM or (G-H) 0.3 μM C-15 (MDM2 inhibitor), (F) 0.01 μM or (G-H) 0.03 μM panobinostat (HDAC inhibitor), or a combination of C-15 plus panobinostat for 24 hours and pulsed with bromodeoxyuridine prior to the end of treatment. Cells were stained with anti-BrdU-Alexa Fluor® 647 antibody, and the percentage of BrdU-positive cells was measured by flow cytometry. Percent inhibition was calculated relative to DMSO control; mean and SEM (n=3) are shown.

Table S1, related to Figure 1. Compounds combined with MDM2 inhibitor C-25 in discovery screen (Screen 1). See Excel file.

Table S2, related to Figures 1-4, 6, 7. *In vitro* biochemical and cellular potencies of the MDM2 inhibitors profiled in combination studies. Experimental methods were carried out as previously described[c].

Inhibitor	Biochemical Potency	Cellular Potency		
	HTRF IC_{50} (nM)[a]	SJSA-1 EdU (10% HS[b]) IC_{50} (nM)[a]	HCT116 BrdU (10% HS[b])	
			p53 WT IC_{50} Transit (nM)[a]	p53 -/- IC_{50} Transit (nM)[a]
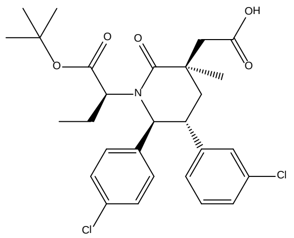 C-25[c]	2.2 ± 0.7	190 ± 60	192[†]	>25,000[†]
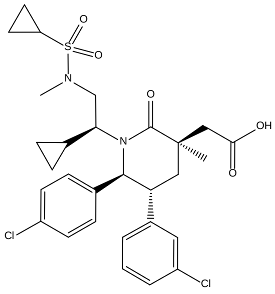 C-15	0.20 ± 0.16	5.3 ± 2.3	3[†]	>25,000[†]
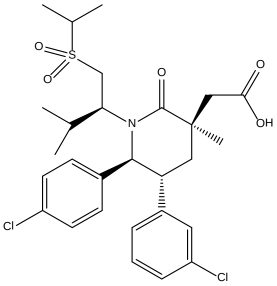 AMG 232	0.6 ± 0.4	9.1 ± 2.8	11.6 ± 7.4	>25,000

[a]Data are reported as the mean±SD (n≥2), except where noted[†]. [b]HS=human serum. [c]Rew et al., 2012

Table S3, Cell Lines, Related to Supplemental Experimental Procedures. Cell line panels for the combinatorial screens with MDM2 inhibitors (Screens 1-3) or PI3K inhibitors are shown. The six cell lines used in triple combination experiments are also noted.

**Cell Line**	**Tissue**	**Combinatorial Screen**				**Triple Combo**
		**1**	**2**	**3**	**PI3K**	
22RV1	Prostate		x	x	x	
A101D	Skin				x	
A172	Brain		x			
A204	Soft tissue		x			x
A2780	Ovary		x			
A375	Skin		x	x	x	
A375 SQ2[a]	Skin					x
A427	Lung		x	x		
A498	Kidney	x				
A549	Lung		x			
ACHN	Kidney		x			
AGS	Stomach	x	x		x	
AsPC-1	Pancreas				x	
BxPC-3	Pancreas				x	
C32	Skin		x	x		
CAKI-1	Kidney		x			
CAL-51	Breast				x	
CAL-54	Kidney		x			
CAPAN-1	Pancreas				x	
CHP-212	Autonomic ganglia		x			
CML-T1	Hematopoietic and lymphoid			x		
COLO-201	Colon				x	
COLO-205	Colon				x	
CW-2	Colon				x	
DLD1	Colon				x	
DOHH-2	Hematopoietic and lymphoid			x		
EOL-1	Hematopoietic and lymphoid			x		
G-361	Skin		x			x
G-401	Soft tissue		x	x		
G-402	Soft tissue	x				
H4	Brain		x			
HCC1143	Breast				x	
HCC1569	Breast				x	
HCC1806	Breast				x	
HCC2935	Lung				x	
HCC827	Lung				x	
HCT-116	Colon	x			x	
HT-1080	Soft tissue		x			
HT-1197	Bladder		x			
HT-29	Colon	x	x	x		
HUTU-80	Small Intestine		x			

Cell Line	Tissue	Combinatorial Screen				Triple Combo
		1	2	3	PI3K	
KS-1	Brain		x	x		
LoVo	Colon		x			
LOXIMVI	Skin		x			
LS-174T	Colon		x			x
LS-513	Colon		x			
MCF7	Breast		x	x		x
MDA-MB-361	Breast				x	
MIA-PaCa-2	Pancreas				x	
MKN45	Stomach		x	x		
MOLM-13	Hematopoietic and lymphoid			x		
NCI-H1155	Lung				x	
NCI-H1299	Lung				x	
NCI-H1650	Lung				x	
NCI-H1666	Lung		x			
NCI-H1975	Lung				x	
NCI-H1993	Lung	x				
NCI-H2009	Lung	x				
NCI-H358	Lung				x	
NCI-H460	Lung		x		x	
NCI-H820	Lung				x	
NCI-H838	Lung				x	
NCI-SNU-1	Stomach		x	x		
PA-1	Ovary	x				
PANC-1	Pancreas				x	
PC-3	Prostate		x	x		
RKO	Colon		x	x	x	x
RPMI-2650	Upper aerodigestive tract			x		
RT4	Bladder		x	x		
SH-4	Skin		x		x	
SJSA-1	Bone	x		x		
SK-BR-3	Breast				x	
SK-HEP-1	Liver		x	x		
SK-MEL-24	Skin				x	
SK-MEL-28	Skin				x	
SK-OV-3	Ovary		x		x	
SNG-M	Endometrium			x		
SUM-190	Breast				x	
SW1990	Pancreas		x			
SW48	Colon		x		x	
SW620	Colon				x	
SW982	Soft tissue			x		
T47D	Breast		x			
U-2-OS	Bone		x			
U-87-MG	Brain	x				
ZR-75-30	Breast				x	

[a]Smith et al., 2009

Table S4, related to Figures 2, S1-S6. Inhibitors used in the combinatorial screens with MDM2 inhibitors (Screens 2, 3) or PI3K inhibitor (PI3K Screen).

Pathway	Target	Inhibitor	Combinatorial Screen		
			2	3	PI3K
MDM2 / p53	MDM2	C-25[a]	x		
		C-15	x		
		AMG 232		x	
PI3K	Pan-PI3K selective	BKM120		x	
		GDC-0941	x	x	
		AMG 511[b]	x		x
	PI3Kα selective	BYL719		x	
	PI3K / mTOR	BEZ235	x		
		GDC-0980		x	
	mTOR selective	AZD8055	x		
		AZD2014		x	
		MLN0128		x	
	AKT	MK-2206	x	x	
		GDC-0068		x	
		AZD5363		x	
MAPK	BRAF	C-1[c-d]	x		
		Dabrafenib	x	x	
		Vemurafenib	x	x	
		P-0685[e]			x
	Pan-RAF	RAF265		x	
		MLN-2480		x	
	MEK	Trametinib	x	x	
		PD0325901	x		x
		Pimasertib		x	
		MEK162		x	
		TAK-733		x	
		GDC-0973		x	
		AZD8330		x	
Intrinsic Apoptosis	Bcl-2 / Bcl-xL	ABT-737	x		
		ABT-263		x	
	Bcl-2	ABT-199		x	
RTK	BCR-ABL, Src, Kit, others	Imatinib		x	
		Dasatinib	x	x	
		Ponatinib		x	
		Bosutinib		x	
		Nilotinib		x	
HDAC	HDAC	Panobinostat	x	x	
		Mocetinostat	x		

[a]Rew et al., 2012. [b]Norman et al., 2012 [1]. [c]Smith et al., 2009. [d]Carnahan et al., 2010 [2].
[e]Plexxikon patent application, WO2007002325 A1.

Table S5, related to Figure 7. Combination ratios of MDM2, MEK, and PI3K inhibitors derived from clustering and search algorithms focused on maximizing either the overall growth inhibition effect or the contribution of the triple combinations over constituent double combinations across 6 cell lines

MDM2i : MEKi : PI3Ki Ratio	Maximize	Cell Line Set
14 : 1 : 5	Effect of triple over double	1 – 6
10 : 7 : 1	Effect of triple over double	1 – 6
15 : 1 : 3	Effect of triple over double	1 – 6
4 : 1 : 1	Overall effect	1 – 6
3 : 1 : 1	Overall effect	1 – 6
6 : 1 : 7	Effect of triple over double	1, 3 – 6
7 : 1 : 7	Effect of triple over double	2 – 6
16 : 1 : 17	Effect of triple over double	1 – 4
7 : 1 : 1	Effect of triple over double	1, 2, 4, 6
36 : 47 : 1	Effect of triple over double	1, 3, 4, 6
46 : 61 : 1	Effect of triple over double	1, 3, 5, 6
21 : 1 : 7	Effect of triple over double	1, 4 – 6
8 : 1 : 7	Effect of triple over double	2, 3, 5, 6
20 : 1 : 8	Effect of triple over double	3 – 6

Cell lines: 1, A204; 2, A375 SQ2; 3, G-361; 4, LS-174T; 5, MCF7; 6, RKO

SUPPLEMENTAL EXPERIMENTAL PROCEDURES

Synergistic Combination Studies with MDM2 Antagonists

Cell lines were purchased from American Type Culture Collection, German Collection of Microorganisms and Cell Cultures, Japanese Collection of Research Bioresources, Public Health England, and the DCTD Tumor Repository (Table S3). Each line was cultured in its recommended growth medium.

For two-way compound combinations, cells were seeded either into 384-well or 1536-well cell culture plates at initial densities ranging from 100 to 7500 cells per well. Sixteen to 24 hours later, compounds were added to the culture plates in a matrixed format, with one agent titrated along the x-axis and the second agent along the y-axis. For all combinations tested in any given cell line, the starting high concentration and dilution factor of each compound were chosen to well-define the curve maximum, curve minimum, and slope over the range of doses selected for the combination screening format. In the initial discovery screen, 6x6 matrices (6-point titrations, including DMSO control) were used. Subsequent confirmation experiments were performed with matrices of 8x8 (8-point titrations) or 10x10 (10-point titrations), with multiple replicates. CellTiter-Glo® Luminescent Cell Viability (Promega) or ATPlite 1step Luminescent (Perkin Elmer) assay kits were used to determine the numbers of viable cells. Luminescence was measured with an EnVision® Multilabel Reader (Perkin Elmer) for each cell line at time zero (V_0) before the addition of compounds, as well as after 72 hours of compound treatment. Growth inhibition (GI) was calculated on a 200-point scale according to the following equations, where V_{72} was luminescence of DMSO control at 72 hours and T_{72} was luminescence of the compound-treated sample: if $T_{72} \geq V_0$, then GI = 100 x (1 – ((T_{72}-$V_{0)}$ / (V_{72} – V_0))); if $T_{72} < V_0$, then GI = 100 x (1 – ((T_{72}-$V_{0)}$ / V_0)). GI values of 0, 100, and 200 represented uninhibited cell growth (i.e. DMSO control), cell stasis, and complete cell killing, respectively. Sigmoidal dose response curves were plotted using a 4-parameter logistic model. Data were analyzed for synergistic interactions using the Chalice™ Analyzer software (Zalicus) which generated synergy scores based on the Loewe Additivity model. In the final screen, the statistical significance of each heterologous combination (AxB) was evaluated by comparing its synergy score to that of its component self-crosses (AxA or BxB) using a two sample Student’s t-test with unequal variance. A heterologous combination was considered synergistic only when its synergy score was statistically greater ($p < 0.05$) than those of both its cognate self-crosses.

Three-way combination experiments were conducted as described above, except with the addition of a third agent to generate 10x10x10 cuboidal matrices. To achieve this, multiple identical two-way combination matrices (10-point titrations) were prepared on separate plates to form the x- and y-dimensions of each cube. Each of the plates received a different concentration of the third agent (or DMSO control) to produce a 10-point titration in the z-dimension. Growth inhibition was calculated as described above. In order to assess the three-way combination interaction for each dose response cube, the Chalice™ Analyzer software was used to assemble the two-dimensional planes of each

experimental cube replicate into a single consensus data cube. Subsequent analyses utilized the highest single subset (HSS) combination interaction model as a reference for the 3-way combination activity. The HSS model compared the observed effect of the 3-way combination to the observed effect of each of the three 2-way combinations at the same concentrations of the inhibitors. The magnitude of numerical superiority of the 3-way effect compared to the highest of the 2-way effect was used to establish the HSS model effect excess at each location within the cube. Additionally, where biological replicates were present, the significance of the HSS excess was tested utilizing a two-tailed t-test. Hierarchical clustering was used to identify privileged ratios that provide beneficial responses across some or all of the cell lines tested. The data utilized for the clustering were each of the points from the individual cell line cubes that demonstrated a significant HSS excess (alpha=0.10). The hierarchical clustering was performed using an agglomerative clustering method with an average distance difference that was based on the ratios of MDM2i:MEKi and MDM2i:PI3Ki to yield clusters which represented similar ratios across the different cell lines. Clustering was performed for all cell lines together, and for each of the permutations of n-1 and n-2 cell lines. Summary statistics were calculated for each level of the resulting dendrograms including the average absolute effect, average HSS excess, average MDM2i:MEKi ratio, average MDM2i:PI3Ki ratio, and fraction of cell lines covered. Privileged ratio clusters were selected based on having 1) average growth inhibitory effect > 140%, 2) MDM2i:MEKi variance < 2.5 fold, and 3) MDM2i:MEKi variance < 2.5 fold. The ratio search was performed across a wide variety of ratios ranging from 1000:1 to 1:1000 for both MDM2i:MEKi and MDM2i:PI3Ki in all permutations for the two ratios to define the three concentrations, with each ratio search directed to a specific objective. The objective of the search was to identify either 1) the maximum overall effect in the triple combination across the cell lines, or 2) the maximum contribution of the triple combination in excess of the double combinations across the cell lines. Three-dimensional isobolograms were constructed by identifying the concentrations of the crossing points of the intended effect level within the full cube, performing a reduction of the dataset to merge data points that were close to each other in space, and finally creating a contour data grid to display the x, y, z coordinates on a log scale (Golden Software).

In vivo Pharmacology

All studies utilized 4-6 week old female athymic nude mice (Harlan Laboratories, Hsd:Athymic Nude-*Foxn1*^{nu}). The mice were housed five per filter-capped cage in sterile housing in an environmentally controlled room (temperature 23 ± 2°C, relative humidity 50 ± 20%) on a 12-hour light/dark cycle. The mice were fed commercial rodent chow (Harlan Laboratories) and received filter-purified tap water *ad libitum*. The mice were individually identified by microchips (Bio Medic Data Systems) which were implanted subcutaneously at least two days prior to the study. RKO cells (5×10^6) were implanted subcutaneously into the right flank in 0.2 mL of equal parts minimal essential medium and BD Matrigel (BD Biosciences). Treatment began on day 7 when tumors had reached ~200 mm^3 (n=10/group). Formulations used were Vehicle 1 (MEK and BRAF inhibitors): 2% HPMC, 1% Tween 80, pH 2.2; or Vehicle 2 (MDM2 inhibitor): 15%

HPβCD, 1% Pluronic F68, pH 8.0. Treatment groups were as follows: 1) Vehicle 1 + Vehicle 2 ; 2) 10 mg/kg C-1 + vehicle 2; 3) 10 mg/kg C-1 + 100 mg/kg AMG 232; 4) 10 mg/kg PD0325901 + Vehicle 2; 5) 10 mg/kg PD0325901 + 100 mg/kg AMG 232; and 6) vehicle 1 + 100 mg/kg AMG 232. Mice were dosed by oral gavage once per day for the next 12 days (Days 7-17), first with Vehicle 1, PD0325901, or C-1, followed by a second dose of Vehicle 2 or AMG 232 one hour later. Tumor volumes, calculated as length × width × height in mm^3, were recorded twice per week. Results were expressed as the mean ± standard error (SE). Statistical significance was evaluated by factorial RMANOVA followed by Dunnett's post hoc analysis for repeated measures using JMP software v8.0 interfaced with SAS v9.1 (SAS Institute, Inc.).

SUPPLEMENTAL REFERENCES

1. Norman MH, Andrews KL, Bo YY, Booker SK, Caenepeel S, Cee VJ, D'Angelo ND, Freeman DJ, Herberich BJ, Hong FT, Jackson CL, Jiang J, Lanman BA, Liu L, McCarter JD, Mullady EL, et al. Selective class I phosphoinositide 3-kinase inhibitors: optimization of a series of pyridyltriazines leading to the identification of a clinical candidate, AMG 511. J Med Chem. 2012; 55(17):7796-7816.

2. Carnahan J, Beltran PJ, Babij C, Le Q, Rose MJ, Vonderfecht S, Kim JL, Smith AL, Nagapudi K, Broome MA, Fernando M, Kha H, Belmontes B, Radinsky R, Kendall R and Burgess TL. Selective and potent Raf inhibitors paradoxically stimulate normal cell proliferation and tumor growth. Molecular Cancer Therapeutics. 2010; 9(8):2399-2410.
